# When the larger objective matters more: support workers’ epistemic and deontic authority over adult service‐users

**DOI:** 10.1111/1467-9566.12964

**Published:** 2019-06-18

**Authors:** Charles Antaki, Joseph Webb

**Affiliations:** ^1^ School of Social Sciences Loughbrough University Loughborough UK; ^2^ Centre for Academic Primary Care Bristol Medical School University of Bristol Bristol UK

**Keywords:** Intellectual impairment, learning disability, empowerment, Conversation Analysis, support, deontics, epistemics

## Abstract

We report on how support workers sometimes over‐ride the wishes of people living with cognitive impairments. This can happen when they are both involved in some project (such as an institutionally‐managed game, a physical journey, an educational activity and so on). The support worker might use their *deontic* authority (to propose, decide or announce future actions) to do things that advance the over‐arching project, in spite of proposals for what are cast as diversions from the person with impairments. They might also use their *epistemic* authority (their greater knowledge or cognitive capacity) to trump their clients’ choices and preferences in subordinate projects. Not orienting to suggested courses of actions is generally interactionally dispreferred and troublesome, but, although the providers do sometimes orient to their actions as balking their clients’ wishes, they usually do not, and encounter little resistance. We discuss how people with disabilities may resist or palliate such loss of control, and the dilemmas that support staff face in carrying out their duties.

## Introduction

Policy about people with a cognitive impairment and/or disabilities often focuses to their right to make choices and have control over their lives (Department of Health, 2001, 2009, 2012, 2015; Glendinning 2008, Leece and Bornat 2006). If they are assessed as needing support and assistance, they qualify for ‘person centred’ care (Fyson and Kitson 2007). However, by receiving support from another person, they give up some agency and control. Doing an activity, or making a choice, becomes a shared and interactionally navigated task. For example while they may choose to do an activity, the person supporting them may have (or at least may display) greater knowledge and understanding about how to *accomplish* it. This often means that enacting an activity becomes a relational exercise between the person with cognitive impairments and those who support them. How does this actually look in practice? If there is virtue in a social relational model that sees disability as an emerging property of actual impairment played out in the social environment (see Thomas 2004a,b), it makes sense to look at everyday interaction between persons with impairment and their supporters as a site in which disabling effects could be enacted. A time when both parties are engaged in choosing, proposing and deciding on an activity is a promising candidate for just that sort of interaction.

Anyone studying interactions between people with some sort of cognitive impairment (e.g. dementia, or intellectual disabilities) and their conversation partners, will be aware of *epistemic* and *deontic* asymmetries – differences in what they have the right to know and in their authority to direct others’ behaviour. The pervasiveness in human interaction of these two rights has been amply shown in work inaugurated by Heritage and Raymond (2005), and by Stevanovic and Peräkylä (2012) respectively. Both can be seen as interactional expressions of power – and acquiescence to it. It is not simply that someone claims the power to direct action or to know best, but the extent to which other interactants accept it (Stevanovic 2018). But epistemic and deontic authority are not equally distributed in society, nor in every interaction; and people with intellectual impairments risk finding themselves at the lower end of the scale *vis‐à‐vis* the person supporting them. There are different kinds of knowledge that can be entailed through talk. People are often treated, and treat themselves, as having the right to know and talk about their own lives and experiences (Heritage and Raymond 2005), or to know how to complete tasks (e.g. cooking, how to choose ingredients, how to get plan a route, which bus to catch, etc.). It is to this latter category of knowledge to which we turn in this paper, and how such activities are attended to and organised in interactions between people with cognitive impairments with their conversation partners.

In the research we report here, we identify ways in which the person with a cognitive impairment's support worker manages an ostensibly joint project by deploying either (in Section 1) deontic or (in Section 2) epistemic authority to over‐ride, ignore or counter the service‐user's proposals for alternative or contributory sub‐projects. Although such bids for action would normally be responded to affiliatively – if only out of the preference for progressivity in interaction (Schegloff 2007, Stivers and Robinson 2006) – we shall see that the support worker implicitly invokes a higher‐order preference for completing the larger objective, at the expense of the subordinate one.

It is important to bear in mind, as we go through the argument, that because we will be concentrating on times when the supporter takes over, the episodes will feature the person with cognitive impairment and the person supporting them in (some degree of) conflict over what to do next, and it will be the case that the supporter's agenda that wins out. This is not to say that the supporters are being overbearing, callous or insensitive; as we shall see, they will have a more overarching objective in mind and may be working towards an end which, although more distant, is in their client's interests. Supporters face a difficult interactional situation – balancing greater needs with local actions‐in‐the‐moment actions which conflict with them. They are often placed in situations in which those two things are difficult to balance. There is a useful discussion of this issue in Pilnick *et al*. (2010), where the authors lay out the contradiction that while trying to respect the ambitions of *Valuing People* (Department of Health, 2001) staff are, structurally, and not as a matter of personal choice, placed in a difficult position where promoting values enshrined in policy, such as choice and independence, comes up against the reality of realistic/possible choices and interdependence. Institutional pressures of time‐keeping, shift‐work and moral imperatives (e.g. fulfilling the duties of one's role and responsibilities towards the service‐user) mean that it is not always feasible to respect a client's wishes, if it meant that a more institutionally mandated objective would not be fulfilled. As we shall see, there are often contingencies above and beyond what the service‐user may be aware of.

## Data

Most of the data presented here were collected as part of a larger study^1^ focusing on how people with cognitive impairments interact together with the people who support them in everyday life (carers, staff, personal assistants. That yielded data from 7 different settings for people with dementia, from five interactions between people with intellectual disabilities and their personal assistants, and from a pottery group for people with intellectual disabilities. For breadth, we also report a case (example 8) from a separate corpus, of service‐users with intellectual disabilities attending a garden‐therapy centre. Informed consent was given for all stages of the research, and all names or identifiable information have been anonymised in the transcripts presented here.

## Analysis

If we are to study the details of how staff and people with intellectual disabilities interact, we shall need a method that that pays very close attention to the details of interaction as they appear in video recordings. Conversation Analysis, the method we report here, is a well‐established approach to the study of talk in interaction, based in typical language, but now with an extensive history of application to interactions involving atypical language use (for an overview, see Antaki and Wilkinson 2012). In the two sections below, we use Conversation Analysis to identify ways in which the person with a cognitive impairment's support worker manages an ostensibly joint project by deploying either (in Section 1) deontic or (in Section 2) epistemic authority to over‐ride, ignore or counter the service‐user's proposals for alternative or contributory sub‐projects (or, where there is a mix, both kinds of authority). We will see how support workers can often find themselves in a bind where there have to manage competing priorities, some of which may be to benefit the person they support, but which require prioritising a larger objective in the future over a locally delivered objective in the present. The data show that in these cases, the support worker may orient to accomplishing an overarching project by: a) dismissing or over‐riding the service‐user's proposals; b) not engaging in side‐sequences they initiate and c) by not including them in the ongoing interaction. In each section we show how this is accomplished.

### Section 1: *Deontic authority – who's in charge*

In what follows, we shall see that in being in charge of a larger overarching project (or taking over that larger project), support workers can treat themselves as having license to reject or ignore the service‐user's proposal for an action on a smaller scale (e.g. some business that is not relevant to the success of the operation, or is not treated as relevant to an overarching activity). They do this in the following examples by exerting their deontic authority to dismiss proposals; ignore side sequences; and not include someone available for, and apparently interested in, an ongoing activity. We give an example and analysis of each of these.

#### a) Dismissing proposals

**Figure 1:**
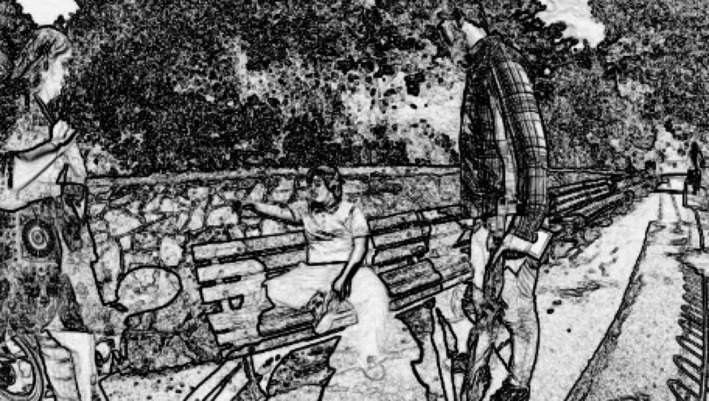
Service‐user Paul (seated) and his personal assistants.

In the following extract, the service‐user (‘Paul’), 19, who has Down syndrome at a moderate level, is accompanied by two personal assistants (hereafter PAs) Robert and Anne, on an excursion through the park after playing mini‐golf. Preceding the extract a decision has been made by Paul to go to town, before returning to his home. His personal assistants propose walking into town in order to catch a bus back to Paul's house. This is the larger framework of what is to come. What we see in the image is the PAs standing, ready to go, but Paul sitting on a bench with a relaxed posture, not in line with the postural ‘readiness’ displayed by his PAs, who stand either side of him (Figure 1).

In the extract, Paul attempts a jocular proposal which is rejected by the PAs, and which they follow up with a direct imperative and subsequent non‐consultation about subsequent actions.

#### Extract 1: Paul and the motorbike^2^


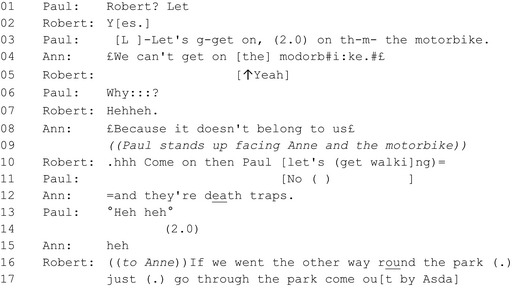


On line 1 Paul produces Robert's name with questioning prosody, selecting him as the intended recipient of his projected upcoming turn. Paul's ‘summons’ is responded to in overlap on line 2 by Robert (‘yes’). Paul then proposes something with a *let's* formula (see Stivers and Sidnell 2016 on *let's* versus other forms of suggestion). The proposal is that they get on the motorbike (lines 3 ‐4), although the non‐seriousness of his proposal is suggested by his half‐smile delivery. This may well relate to the fact that Paul has just chosen to return home; the motorbike being a joking way of proposing transport to complete the action he initiated. Indeed, as Stivers and Sidnell show, *let's* forms imply some disjunction between the now‐proposed activity and what has already been established (whereas the form *how about…* does not). One way or another, the joking proposal is rejected by Anne in smile‐voice (line 4), despite not being named as the intended proposal recipient.

Following this, Paul uses ‘why’ (line 7) to question the basis of his rejected proposal, and in so doing prolongs or perhaps upgrades his joke, requiring a further response from the PAs, and hindering progress. His insistence on treating what would be conventionally a non‐serious suggestion as worthy of debate puts the PAs at something of a disadvantage in responding; in a sense it is a tease, to which the typical response is some sort of dismissal (see Drew on what he calls ‘po‐faced’ responses to teasing; Drew 1987). This is indeed what happens: Anne does not engage with the humour of the tease but instead with its surface seriousness, dismissing the suggestion firstly on the grounds that the motorcycle is not their property, and secondly on the grounds of danger. The other PA could also respond in the same serious register, or could, alternatively, take Paul's side and prolong the tease further by joining in with the playfulness of the proposal; but in fact he does neither, choosing instead to cut away from the topic entirely and issue a direct imperative (line 10). He draws on his deontic status to bypass Paul's joking suggestion, and to propose and direct what happens next (let's get walking’). Thereafter (line 16 and following, not shown), the PAs discuss the route between themselves, and Paul follows behind as they walk on.

#### b) Not engaging in side‐sequences

In the prior extract we have seen how a proposal/suggestion can be overridden, or not attended to, in the service of completing an overarching activity that the disabled person had initiated. The completion of an activity, even if it is one the person chose, can therefore give license to disattend to turns which are either non‐relevant to the activity or would otherwise work against its completion. In the following extracts we will examine a related phenomenon; how side sequences not relevant to the completion of an activity the person with disabilities has chosen can also be disattended to. A side‐sequence refers to a break in contiguity via an interruption of an ongoing activity (Jefferson 1972), and in the following sequences often involves the introduction of a new interactional project (see Schegloff and Sacks 1973) unrelated to a jointly engaged in overarching activity.

The supporter not attending to a side sequence initiated by a person with cognitive impairments was common throughout our data set when an overarching project was being enacted. The following extract involves Melinda (a woman in her early 50s with acquired cognitive impairment affecting everyday functioning and intellectual ability) and Lucy, who Melinda has employed from her care budget). Lucy is engaged in dismantling chairs that Melinda recently bought, ready to be returned because Melinda was not satisfied with their quality.

We join the extract as Lucy is engaged in taking the screws out of the chairs in order to fit them in the packaging. After a lengthy gap, Melinda walks to the window and makes an observation about the weather.

#### Example 2: Melinda and the chairs


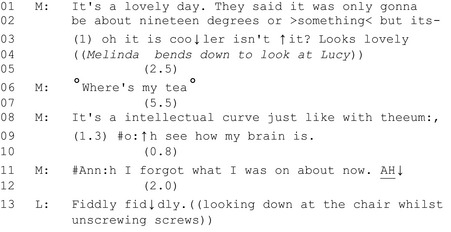


**Figure 2:**
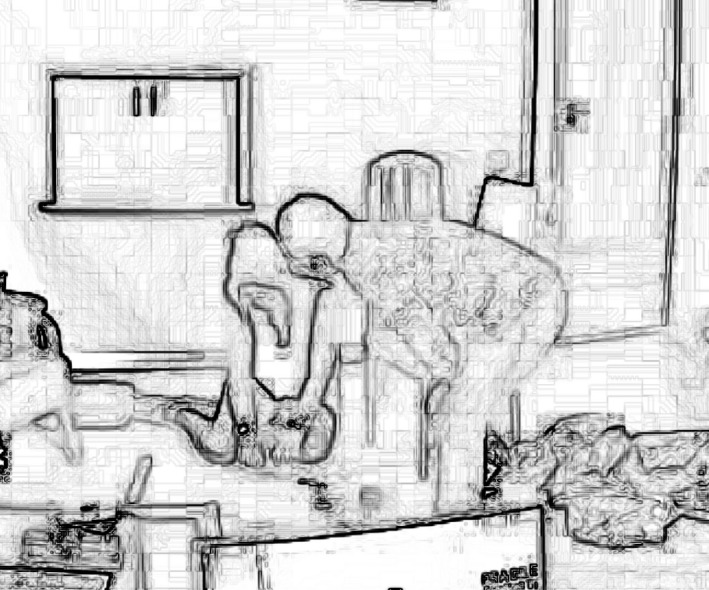
Lucy squatting on the floor disassembling a chair – Melinda leaning over her.

On line 1 Melinda produces an assessment of the weather whilst looking out of the window (‘*it's a lovely day’*) which trails off to a gap of 1 second, strongly projecting an affiliative agreement (Pomerantz 1984) from Lucy. That does not come. Melinda then produces an ‘oh‐prefaced’ formulation plus tag question, and a reissue of the ‘lovely’ assessment, whilst looking at Lucy; indeed, Melinda walks right up to Lucy, bends down and makes the assessment a couple of inches away from her face, whilst staring directly at her (see the image in Figure 2). This is a palpable upgrade, in both verbal and non‐verbal modalities, of the strength of the expectation that Lucy should respond (Stivers and Rossano 2010). However, Lucy remains occupied with the task of packing up the chairs.

There is a 2.5 second gap where Lucy could step into the second position turn space that Melinda has left for her. Upon receiving no reply, Melinda stands up and extends her turn by starting a new sequence with ‘where's my tea’. Uttered at low volume, this seems to be an example of Goffman's (1981), perhaps glossing over Lucy's failure to respond; indeed Lucy continues to pack up the chairs. After an extended silence of 5.5 seconds (line 7), Melinda begins yet another topic of conversation where she reflects upon the process of buying (and returning) items from the Internet (lines 8‐9). At this point she signals a self‐repair, with a word‐search token (*thee‐um*). This, now, may be a chance for Lucy to take part. Oelschlaeger and Damico (2000) in their study of word searches amongst people with aphasia show that the action of searching for a word can be a collaborative activity, whereby co‐interactants can use various strategies to help the person searching for the word. Although stepping in too early is dispreferred (e.g. before the speaker has had sufficient time to retrieve the word), this is an opportunity for Lucy to aid Melinda in her word search, or at the very least to look at her as she attempts to make a comparison between their current situation and a previous occurrence. Instead, Lucy maintains concentration on completing the task.

After a gap (line 10), Melinda then launches into what can be described as ‘troubles telling’ (Jefferson 1988) where her inability to remember a word occasions a subsequent formulation which invites Lucy to react to, or sympathise with, her outlined cognitive difficulties (‘*oh see how my brain is’*). Upon receiving no reply, Melinda's turn extension (line 11) once again foregrounds her cognitive difficulties (‘*Ah I forgot what I was on about now. Ah!*’). The utterance is prefaced and ended with ‘ah!’, emphasised particularly at the end of the sentence, expressing exasperation at not only Melinda's inability to find a word she had forgotten, but her sense of her own cognitive issues. One may expect a fitted second pair part to empathise or sympathise with exasperation and troubles telling (Jefferson 1988), but Melinda is again met with silence. When Lucy does finally speak, it is not as a fitted second pair part to Melinda's extended turn, but as a comment on the difficulty of unscrewing the chairs that her eye gaze remains firmly fixed upon (‘*fiddly fiddly’*).

This whole side‐sequence (Jefferson 1972) initiated by Melinda is not relevant to the accomplishment of the overarching activity of packing up the chairs, but introduces a new interactional project (Schegloff and Sacks 1973); namely making small talk on a subject about the weather, and latterly about Melinda's inability to recall certain words and her ‘troubles telling’ about this difficulty. Whilst irrelevant to the joint task in which they are both engaged, such an action in first‐position is typically (though not always) treated as an ‘assessable’ by the intended recipient in second position (Stivers 2012). As in the previous extract, as the first half of an adjacency pair, a normative obligation is placed upon Lucy as co‐interactants to perform a type fitted response (Sacks 1992, Schegloff 1968). However, responding appropriately may come at the expense of failing to fulfil the institutional obligations of Lucy's role; a task that Melinda has herself chosen. Here we see again how the supporters carrying out an activity (even one chosen by the person with cognitive disabilities) licenses their non‐responses. Melinda created several opportunities through a variety of actions for possible spaces for Lucy to produce a second pair part, such as her initial asserted assessment about the weather which provided for the relevance of, and typically engender second assessments; but Lucy pressed on with the overarching task.

#### C) Non‐inclusion

In the previous examples, the support worker's authority over the running of the project at hand allows them to, or manifests itself in, respond minimally or not at all to a proposal or a side sequence initiated by the person with intellectual disabilities. The example below shows an occasion on which a service user with dementia, although signalling her receptivity to possible engagement via body posture and eye gaze, is entirely left out of an activity.

#### Extract 3

In a ‘memory cafe’ (Harmer *et al*. 2017) for people living with dementia and their family members, time is allocated for group games, led by a facilitator. Pat, who is in her early 80s and has dementia, (seated on the left of the picture) is doing a quiz with her visiting daughter Mel and her ‘dementia navigator’, Anne, on the right. We join the group as the engage in a quiz about famous crimes and criminals throughout history. The extract begins with the group discussing a possible answer to a question about the location of a famous historic prison. Lynne, a service‐centre organiser, walks over behind them and leans over the table between Mel and Anne. All three pore over the quiz sheet.


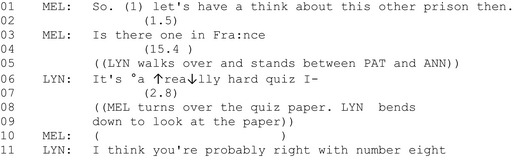


**Figure 3:**
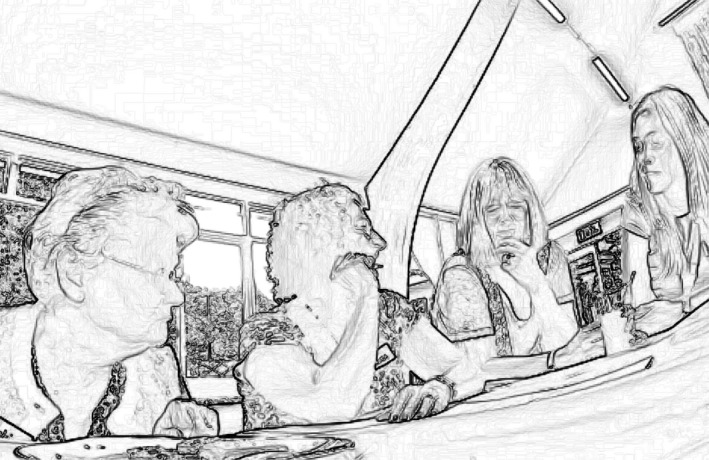
Pat (on the left) with the other quizzers.

**Figure 4:**
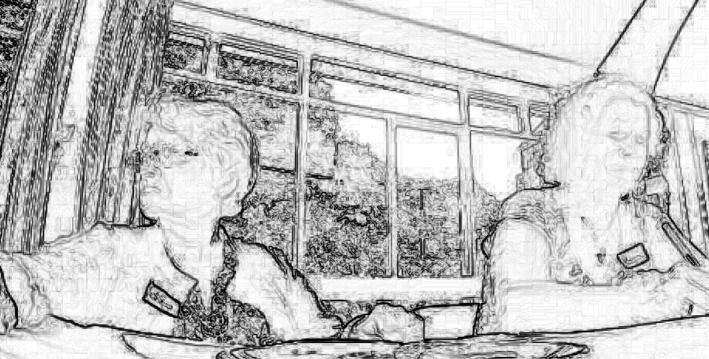
Pat (left) slumps back and turns away from the quiz group.

Figure 3 shows Pat in the leaning‐in posture that she adopts throughout the next few minutes, and from which she occasionally looks up in apparent readiness to be addressed by another member of the group. No‐one does. There's no need to show the entire transcript, since at no point does anyone around the table – Pat's daughter, Lynne the dementia navigator, Lynne the service manager – address Pat or look towards her. Eventually she simply slumps back in her chair and turns her head away (Figure 4).

Pat's slumping action has much in common with Clift's ‘visible deflation’ (Clift 2014), characterised as a physical display of exasperation. However, unlike in Clift's analysis, Pat's slump does not appear a response to a *specific* prior turn, but rather a cumulative disengagement borne out of a response to her fellow‐quizzers’ numerous prior turns that exclude Pat from recipientship. Pat can in fact speak and engage in conversation. Indeed, on the same data collection day, she was involved in a different type of quiz – equally hard, but the other participants made sure she was included. Here, though, the other participants keep the quiz sheet too far away, and effectively ignore her as they prioritise getting the answers right over including her in the game. Where, as here, the drive for progressivity (getting the questions done and finishing the quiz) is the overall activity to which participants turns at talk orient, it can work against the ostensible therapeutic purpose of this type of quiz: facilitating social interaction. Perhaps we should say that the element of direction (the facilitator taking charge of who plays, who gets a turn) – is inextricably mixed with the matter of (implied) competence (who knows, who might have the answer). So the episode also has a strong flavour of *epistemic* asymmetry – the supporters treating Pat as not able to participate in the quiz due to the difficulty of the questions – and we turn to still clearer forms of that kind of asymmetry next.

### Section 2: *Epistemic authority – who knows better*

The following examples demonstrate that although the support worker's directive, talk‐oriented authority is clearly in evidence, it is based on the support worker's claimed epistemic knowledge and rights. What they know, and how they think, about the overarching task they are engaged in puts them in a position to over‐ride service‐user's needs *in the moment*, often in the service of a possible longer‐term benefit. We see the same patterns as above, that is a) dismissing or over‐riding the service‐user's proposals, b) not engaging in side‐sequences they initiate and c) not including them in the ongoing interaction.

#### A) Dismissing or over‐riding the service‐user's proposals

In the example below, Bonnie, a woman in her early thirties with mild intellectual disability and cerebral palsy, is in a supermarket with Padma, her PA, shopping for the makings of her evening meal. Bonnie has indicated (outside the supermarket, before this episode starts) that she intends to cook a stew from a recipe she decided on earlier which would involve some cut of meat.

#### Extract 4


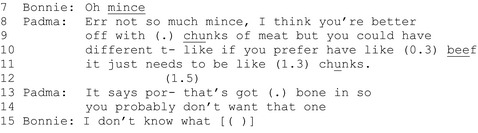


Having selected a stew recipe previously, Bonnie sees mince on the shelf; her use of the turn initial particle, ‘oh’ precedes the verbal action of noticing the mince (Heritage 2017), whilst the exaggerated prosodic delivery of mince brings the object to the attention of the PA, verbally displaying her intention to buy mince for the recipe. The PA's response is prefaced with ‘err’, typical of dispreferred responses in second‐position (Pomerantz 2006) and orients to the dispreferred nature of the upcoming imposition of choices. The PA continues with an epistemically marked utterance in second position, ‘I think you're better off with chunks of meat’. Coming as it does as the second part of an adjacency pair, it serves to disagree or otherwise question the interactional move made by Bonnie (Kärkkäinen 2003). The full utterance (from line 10 onwards) seems designed to make future choices conditionally relevant to the modifying terms the PA has suggested. Here, the PA draws on her epistemic status to ‘know best’ what the recipe requires and to therefore guide Bonnie in what she ought to choose – Padma is even able to claim some access to what would suit Bonnie: line 13‐14: ‘that's got bone in so you probably don't want that one’.

We skip forward a minute and 10 seconds in the transcript. Several more attempts at choosing an adequate meat in first position by Bonnie are met with dispreferred second pair parts by the PA for reasons of price, suitability to the recipe, and similarity to a recently cooked meal. At line 60 the PA picks the meat herself (line 60 and 61), giving the rationale that pork was in the original recipe Bonnie chose. After having her own choices in first position repeatedly questioned, Bonnie assents to her PAs choice of a suitable meat for the recipe (line 66).

#### Extract 5: B. and the steaks


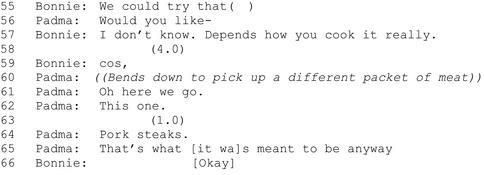


After all, Bonnie had evidenced some uncertainty at 57, so P volunteering a new choice does not look out of place. However, B's display of uncertainty, coming as it does after having multiple choices questioned due to their suitability, is perhaps not surprising. The PA has made drawn on her epistemic status (Heritage 2012b) as having the right to question or otherwise problematise Bonnie's choices. Indeed, at each stage the PA adopts a stance of having greater knowledge – whether she does or not – positioning herself as a more knowledgeable guide to Bonnie's choices. She claims licence to know what Bonnie is better off with buying.

Here then we see that whilst a service‐user can make the choice over what recipe to cook, that entails multiple subordinate choices (choosing where to shop, choosing what ingredients, etc.) that go towards fulfilling the overarching activity. Though Bonnie is nominally in control of the larger choice of what to cook and eat, her personal assistant's greater knowledge – her claimed epistemic authority – allows her to guide, challenge, or override Bonnie's choices. Padma is in the difficult position of either respecting Bonnie's choice in the moment or using her greater knowledge to teach Bonnie about appropriate ingredients, possibly for a longer term benefit.

#### b) Not engaging in side‐sequences they initiate

**Figure 5:**
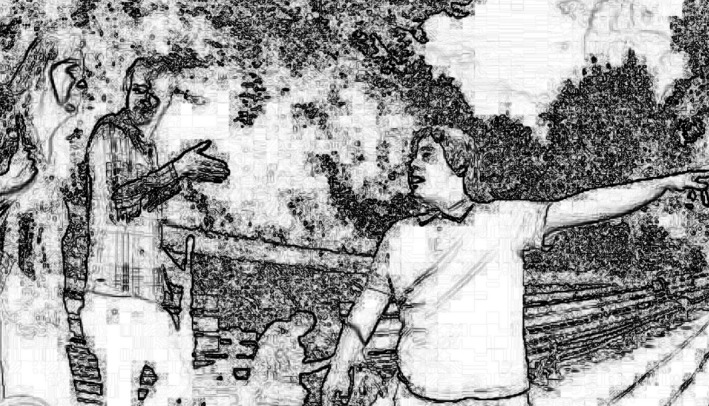
Paul points to the tennis courts.

**Figure 6:**
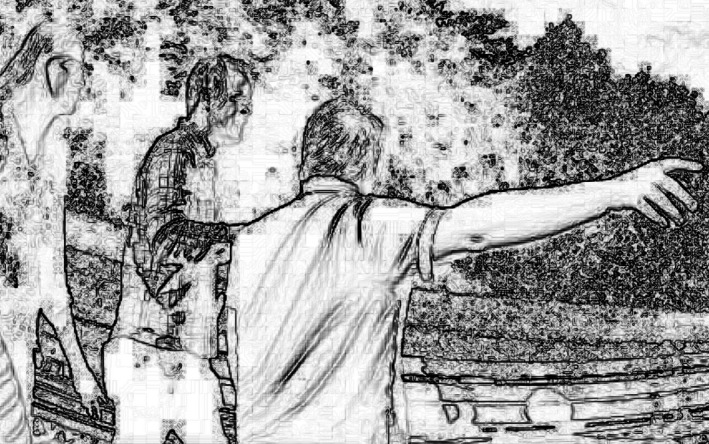
Paul points as the PAs walk on.

In our analysis we noticed that epistemic authority over a particular task (i.e. knowing best how to complete the overarching activity) could result in side sequences initiated by the person with cognitive disability being left out of the interaction altogether, and having to force their way back into the ongoing talk. The example below follows on immediately from extract 1, where Paul and his PAs are walking through the park. As we saw, Paul had originally made the decision to return home, and after reacting ‘po‐faced’ to a teasing suggestion that he take a motorbike, his PAs treated this as necessitating their joint planning about the optimal route to take (joint planning is seen as highly desirable in the personalisation of services; see, for example, Gridley *et al*. 2014). We join the extract now as the PAs jointly formulate a plan for getting home. As in the previous example, Paul has to be tenacious and repetitious to have his bid to a topic‐initiation (a sensitive matter at best of times – see Button and Casey 1988) attended to by his co‐interlocutors. It should be remembered that what all three of them are doing is at the behest of Paul, who has elected to go to town first before heading home by bus); so, nominally, progress ought to be at his pace (Figures 5 and 6).

#### Extract 6: Paul and the tennis courts


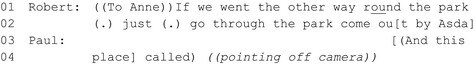



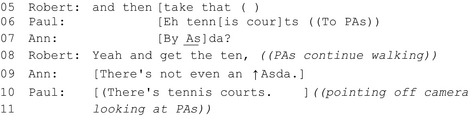



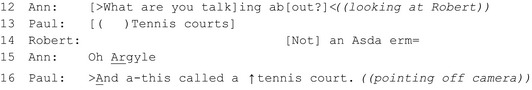


**Figure 7:**
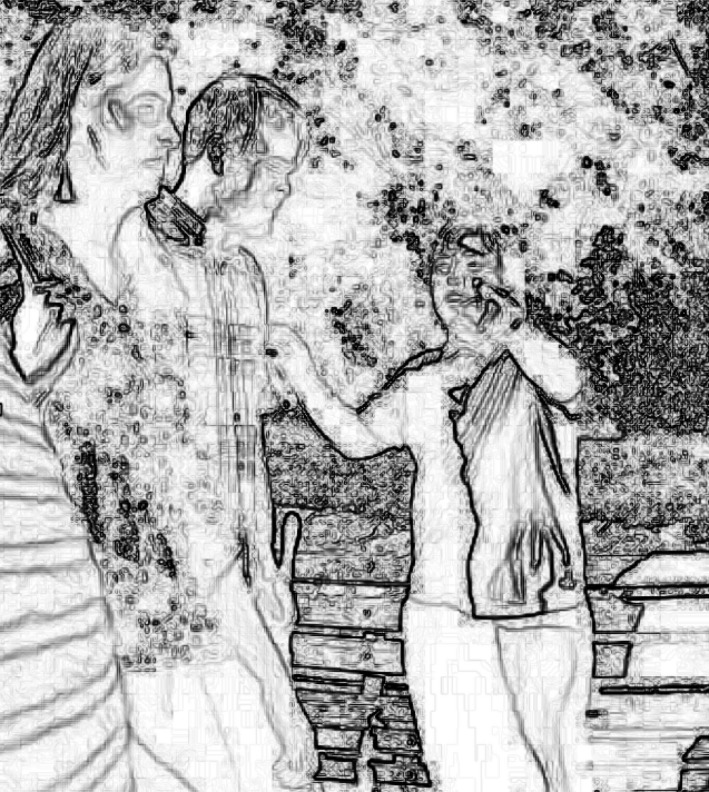
Paul positions himself in front of PA Robert and points as the PAs walk on.


The extract begins where Example 1 had left off, with Robert talking to Anne and suggesting a route to take in order to get to town. While the PAs discuss the route between themselves, Paul tries three times to direct their attention to the tennis courts situated to their right as a topic of conversation, but none of his utterances are responded to by the PAs as they continue to talk between themselves. Paul positions himself alongside, and sometimes behind, the PAs as they walk along, but finally he upgrades his demand for reply by manoeuvring himself almost direct in front of Robert (see Figure 7). It is only on this, his fourth attempt (‘*and this place is called tennis courts’)*, that Robert responds to his repeated utterances.

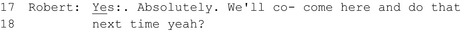


Robert's response (lines 17‐18) is a finely‐constructed amalgam of new‐receipt and proposal acceptance. The initial *yes*: is a pronounced with a terminal intonation; it is a place where his turn might have ended – but if it is an assent, it is an assent to what is ostensibly a mere news‐announcement (that the place is called ‘the tennis courts’); but to assent to a news‐announcement is to imply that it is already known. Then Robert adds the increment *absolutely*, amplifying the effect. So far in his turn, then, Robert has treated what Paul has said as an assertion of an already‐known fact. That would be consistent with taking it as the basis for topical development (e.g. by asking Paul if he'd been there, or likes tennis, and so on). But in the remainder of his turn (*We'll co‐come here and do that next time yeah?*’), Robert uses this now established fact about what the place is called as the basis for something quite different from topic‐development. He shifts to orienting to Paul's turn as a proposal for action, implying that what Paul had wanted was not to talk about the place, but to go there now. That brings it into Robert's deontic domain: his institutional authority to take charge of the overall management of the current trip (and, by implication, future trips to come). By Robert's combination of already‐known (epistemically prior) news‐receipt, and redirection towards immediate action, Paul's topic‐proposal evaporates away.

#### C) Non‐inclusion in the ongoing interaction

**Figure 8:**
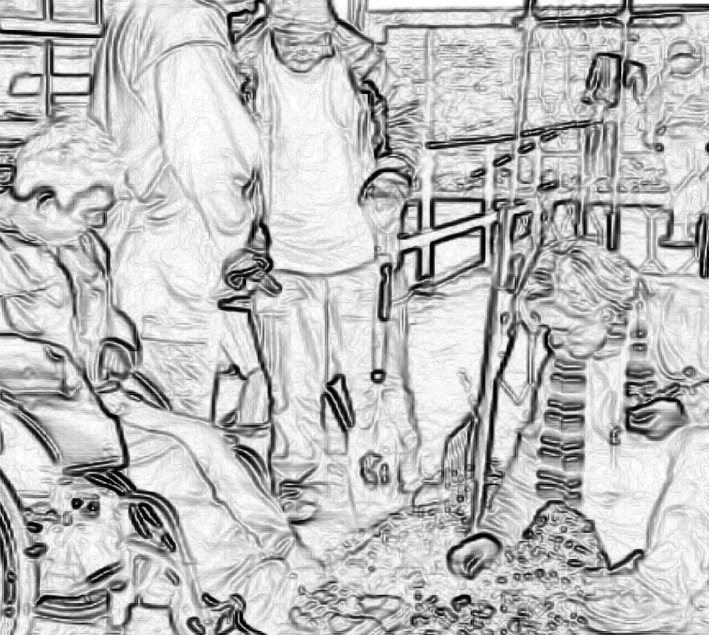
Cameron (on the left, in the wheelchair) and Deb (kneeling, right).

In the following extract, a horticultural therapist is explaining to a group of service users with intellectual disabilities about taking care of herbs. She leans down and describes the state of a patch of thyme. Cameron (in the wheelchair, on the left of the photo; his disability is unrecorded) directs his eye gaze towards the horticultural therapist's hands as she touches the thyme (Figure 8).

#### Extract 7: Cameron and the plants


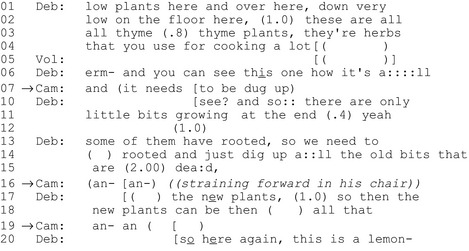


On lines 1‐ 4 we see Deb, the horticultural therapist using deictic gestures (‘*low plants here and over here…’*) in order to direct the clients’ attention to the relevant area's plants, adopting a stance of greater knowledge, relative to another (Heritage 2012a,b) in ‘doing informing’ to the group. This has some parallels with Goodwin's concept of professional vision (1994) as she directs the group to see particular aspects of the plant (a*nd you can see this one how it's all very woody*); again, taking a stance as she directs the group to see what she sees.

After a gap of 1.5 seconds, Cameron produces an utterance which is grammatically latched with the conjunction *and*, and which provides a sensible completion of the therapist's implied impending instruction: *you can see this one how it's all very woody / and it needs to be dug up*. Interestingly, Cameron's turn completing the therapist's utterance adopts the same high epistemic stance; a declarative assertion. However, whilst Cameron's turn orients to the prior therapists turn explicitly grammatically and topically, his turn is not oriented to by the therapist. Indeed, she overlaps it at a point where there is no natural transition place, marking her overlap as competitive, rather than affiliative (Schegloff 2000). A few lines later, Cameron again attempts to engage with the interaction, this time with less well articulated turn–initial particles (line 16), but again the therapist presses on with her own explanation. In both cases we may read what has happened as being to some degree a matter of her deontic authority in the task, but also and perhaps more suitably here, her greater horticultural expertise and epistemic authority.

Cameron here is doing more than simply agreeing with the informing practices of the therapist (and therefore claiming understanding); his turn on line 9 is what Sack's terms ‘exhibiting understanding’ (1992, p.252) as he anticipates the projected future action and completes the therapist's utterance. However, in the very act of informing the group, the therapist also enacts a teaching, or informing, role. As Pomerantz (2003) and Pomerantz *et al*. (1997) observe, the activity of teaching inherently defines the one being taught as not fully knowledgeable or competent. Although Cameron attempts to assert his own knowledge, or at least exhibit understanding of the imparted information, here the therapist draws on her epistemic status – as teacher and plant expert – to not attend to his utterances. Again, an overarching institutional objective is in conflict with a local objective; will engaging in a conversation with Cameron, or allowing him to interject in non‐transition relevant places, come at the cost of her institutional aim of teaching?

## Discussion

We set out in this article to identify how it was that a support worker might have licence to override the wishes of the person they're supporting, even when the activity is one that has been chosen by them. We showed how the support worker's (claimed or actual) epistemic and deontic authority can allow them to: dismiss the person's in‐activity proposals; not to engage with their side‐sequences; and even to ignore their very presence. All this was licenced by the overarching – usually institutional – activity that was underway, be it a shopping trip, an educational project or a journey from one place to another. Here the supporter could claim to have the authority to direct the steps of everyone concerned towards the larger objective, and to know better how it was to be achieved. Subordinate activities could be suppressed, or left to fizzle out.

On a micro‐level, from the point of view of the supporter, one operative consideration may be the matter of the agreed ownership of the overarching activity. Rossi (2015) shows that having first established an activity as a *joint* commitment determines the linguistic forms one person may use to successfully recruit the other for co‐operation within that activity. Thus, in his data from Italian households, how one person got co‐operation from the other person was realised in linguistically distinct ways. If the action was part of a more encompassing project (such as jointly cooking a meal) to which the parties had already established a shared commitment, then a certain form (the imperative) could be used in requests for help. But if the action was seen as merely personally motivated, a different form (the interrogative) was used. Our data are a step above the micro, but lend themselves to the same kind of interpretation: that the degree of (perceived) commitment to a joint, overarching activity may decide how the supporter deals with the person with disabilities when they propose an action that may deviate from it.

Perhaps a still more powerful consideration for the supporter is their judgement of what might be in the best interests of the person they are supporting, and their own contingencies in realising it – a consideration that is often a source of concern for supporters (Schelly 2008, Williams 2011). This is perhaps clearest in our Example 1, where service‐user Paul has (apparently jokingly) suggested riding a motorbike. The fact that Paul has two personal assistants, rather than the more usual one, may signal previous instances of challenging behaviour. If so – and perhaps anyway – the personal assistants’ over‐riding of his suggestion, and unilateral decision to simply start walking, will have been in Paul's best interests.

Although such decisions whether or not to accede to the person's wishes are taken on a moment‐to‐moment basis, situations such as Paul's remind us that the supporters are not wholly free to act in whatever way they chose; they have institutionally mandated roles, and objectives to fulfil. Support workers always face the dilemma of care versus control (for a recent overview, see Sims and Cabrita Gulyurtlu 2014); here we have evidence of the dilemma emerging in specific interactional practice. The support worker has a duty to reach the given goal (to deliver the person with disabilities home; to get the pottery piece shaped sufficiently well to be fired; and so on), but in doing so, against various contingencies, they may find themselves inadvertently helping to perpetuate asymmetry. In example 2 we saw above, one could object that the PA, although busy packing up the chairs, could easily answer Melissa when she makes small talk. But the internal evidence from the video reveals that the PA is against time, doing a difficult (or at least taxing) physical task which risks overrunning her paid hours. Or in Pat's case – she was left out of the quiz game, Example 3 – her supporters may have judged that it would be a kindness not to tax her with questions she could not answer. Such countervailing objectives are common in supporting people with intellectual disabilities in the community, as Williams has extensively catalogued (Williams 2011), and have to be managed locally (or interpreted retrospectively – see Webb *et al*. 2018) – and sometimes, as in Pat's case, they may be decided one way on one occasion and another on a different one: the very same day, she was successfully included in a different kind of quiz possibly because the quiz was based on answers from which she could draw on her own experiences.

Do the people with cognitive impairments in our data resist, or complain, when balked? Not overtly. But it is worth noting that they are alive to the likelihood that their wishes may turn out to be frustrated. It is noticeable that they sometimes to cast their proposals in a form from which they may easily retreat. One way of so doing is to put them in what Curl and Drew (2008) term a low entitlement / high contingency form – that is to downplay their own rights in the matter, and acknowledge the difficulties that their action might cause their supporter. This is perhaps clearest in Example 2, where service‐user Melinda explicitly downplays her own competences (*oh see how my brain is*) in the face of her personal assistant's non‐take‐up of her projected action. Further on in that conversation (not shown here), Melinda explicitly orients to Lucy's contingencies when she says to her *don't worry it's my my f‐ my responsible‐ (.) you know don't worry, if I lose the money it's only thirty quid*. Such downplaying is part of any life, but it is possible to see what Melinda does as a realisation of her own identity as a disabled person (a ‘toxic identity’, according at least to Rapley *et al*. 1998).

The other way that people with impairments may immunise themselves to some degree against rejection and frustration is by casting their proposed actions unseriously. That is most obvious, in the examples we've shown here. in the actions of Paul in Example 1, where his suggestion of travelling by motorbike is both unserious in content, and delivered with a half‐smile. The assistants may have gone along with the joke, but if not (and they don't) then their rejection is less face‐threatening than had it been a serious alternative. Further along their journey home, in an episode not shown here, Paul and his assistants have been walking for a time. In a further exercise of their deontic authority, they have taken the lead physically, and Paul is now out of sight behind them. While the assistants are forging ahead, Paul runs off and hides behind some stairs, only to emerge laughing when (easily) found. Running off and requiring the assistants to hunt for him is a dramatic bid for attention, but casting it as a cheeky, rather than a serious, assertion of his rights preserves face against possible admonishment.

In sum: people with cognitive impairments’ low epistemic status, and dubious deontic authority, always put them at risk of exclusion. What we have shown here, with a small selection of what we think are telling examples, is that this is true even when the activity at hand has actually been chosen by the person, or is explicitly in their interests. In addition, support workers may find themselves in difficult situations where they have to prioritise an overarching project over a local one, both of which may have been instigated by the person with a cognitive impairment. That overarching activity can license support workers to decide the dilemma of support versus control on the side of the latter, at the expense of the former.

**Table:** 

Transcription Symbols	
(.)	Just noticeable pause
(.3), (2.6)	Examples of timed pauses, in seconds
word [word] [word]	Square brackets aligned across adjacent lines denote the start and end of overlapping talk.
.hh, hh	In‐breath (note the preceding fullstop) and out‐breath respectively.
wor‐	A dash shows a sharp cut‐off
wo:rd	Colons show that the speaker has stretched the preceding sound.
(words)	A guess at what might have been said
()	Talk too unclear to merit even a guess.
word= =word	The equals sign shows that there is no discernible pause between two speakers’ turns or, if put between two sounds within a single speaker's turn, shows that they run together
word, WORD	Underlined sounds are louder, capitals louder still
£word£	Delivered in a ‘smile’ voice
#word#	‘Creaky’ voice
°word°	Material between ‘degree signs’ is quiet
>word word< <word word>	Inwards arrows show faster speech, outward slower
↑word	Upward arrow shows upward intonation
↓word	Downward arrows shows downward intonation
wo(h)rd	(h) shows that the word has ‘laughter’ bubbling within it
((*gruff voice*)	Attempt at representing something hard, or impossible, to write phonetically
→	Analyst's signal of a significant line

## Notes

1 Those data were collected as part of research about disabling practices, funded by the Economic and Social Research Council, grant no. ES/M008339/1.

2 The Jefferson notation conventions (Jefferson 2004) in the transcript are those commonly used in Conversation Analysis. For a description, please see the glossary in the Appendix
